# Epidemiology of major reproductive health problems and associated infectious diseases in commercial and smallholder dairy herds in North Shewa, central highlands of Ethiopia

**DOI:** 10.3389/fvets.2025.1544789

**Published:** 2025-06-27

**Authors:** Aweke Engdawork, Haileleul Negussie, Awoke Melak

**Affiliations:** ^1^Ethiopian Biodiversity Institute (EBI), Addis Ababa, Ethiopia; ^2^College of Veterinary Medicine and Agriculture, Addis Ababa University, Bishoftu, Ethiopia

**Keywords:** bovine brucellosis, dairy herds, infectious bovine rhinotracheitis, North Shewa, reproductive health problems, seroprevalence

## Abstract

**Introduction:**

Reproductive health problems are disorders of the reproductive system and are the most common cause of economic losses in the dairy industry. Despite the widespread occurrence of reproductive health problems and infectious diseases, their epidemiology is little known in Ethiopia.

**Methods:**

A cross-sectional study was conducted to identify major reproductive health problems, infectious reproductive diseases, and risk factors in commercial and smallholder dairy herds in North Shewa, central highlands of Ethiopia. Blood samples were collected from randomly selected 142 dairy herds and 511 animals and serologically examined with ELISA for IBR and CFT for brucellosis. Two years of retrospective data were collected to identify major reproductive problems in dairy herds.

**Results:**

This study indicated overall seroprevalence of IBR in 85.21% (95% CI: 78.28–90.21%) dairy herds, while bovine brucellosis was prevalent in 3.52% (95% CI: 1.46–8.26%) herds. The most prevalent reproductive problems in dairy herds were abortion (27.46%; 95% CI: 20.68–35.48), retained placenta (33.80%; 95% CI: 26.42–42.06), repeat breeding (40.14%; 95% CI: 32.32–48.50), anoestrus (37.32%; 95% CI: 29.68–45.66), and calf mortality (29.58%; 95% CI: 22.58–37.69). Herds infected with IBR were more at risk of abortion (OR = 8.34; 95% CI: 1.91–76.96; *p* = 0.006), retained placenta (OR = 8.61; 95% CI: 1.04–70.89; *p* = 0.045), repeat breeding (OR = 3.16; 95% CI: 1.82–12.23; *p* = 0.009), anoestrus (OR = 6.63; 95% CI: 1.28–34.38; *p* = 0.024), and calf mortality (OR = 4.05; 95% CI: 1.81–20.32; *p* = 0.008). Brucellosis exposure increased abortion by 21 (*p* = 0.037), retained placenta by 19 (*p* = 0.003), and anoestrus by 12 (*p* = 0.002) times. Herd size and breeding methods were significantly associated with abortion, repeat breeding and calf mortality; while replacement strategies and bull-sharing were significant factors of retained placenta and anoestrus (*p* < 0.05).

**Conclusion:**

This study demonstrates higher occurrences of reproductive problems and circulating infectious diseases in commercial and smallholder dairy herds. Thus, integrated disease control measures such as vaccination, biosecurity, herd screening, and sound management should be practiced to control reproductive health problems and promote the growing dairy production in North Shewa in the central highlands of Ethiopia.

## 1 Introduction

Reproductive health problems are among the most common disorders in dairy cows, affecting reproductive efficiency and productivity, and resulting in considerable economic losses in the dairy industry (1). Reproductive efficiency is one of the most important and desired characteristics of dairy cattle production. Effective dairy production depends on optimal reproductive performance and productivity for the success of dairy operations (2). Reproductive problems are disorders of the reproductive system that prevent or restrict estrus, conception, pregnancy, calving, and productive efficiency. The most common infectious causes of reproductive health problems include infectious bovine rhinotracheitis (IBR) (3), leptospirosis (4), bovine brucellosis (5), campylobacteriosis (4), bovine viral diarrhea (BVD) (6), and trichomoniasis (4).

Reproductive disorders have substantial and immediate impacts on the reproductive performance of dairy animals (7). The major reproductive problems in dairy cows include abortion, dystocia, stillbirth, retained fetal membranes (RFM), metritis, pyometra, uterine and vaginal prolapse, anoestrus, and repeated breeding (8, 9). Reproductive problems can be classified into three categories according to their phase of occurrence: pre-gestation, gestation, and post-gestation. The reproductive problems, such as anoestrus and repeat breeding, occur during the pre-gestation phase. Abortion, dystocia, and stillbirth occur during gestation. Reproductive disorders, including retained fetal membrane, uterine and vaginal prolapses, and metritis, occur during the post-gestation phase (10).

The occurrence of most reproductive problems is closely associated with infectious reproductive diseases, primarily infectious bovine rhinotracheitis and bovine brucellosis (3, 11). IBR and bovine brucellosis are among the most significant reproductive diseases in cattle, which cause various reproductive disorders and infertility issues, particularly in commercial and smallholder dairy production systems. These diseases lead to reduced reproductive performance, poor productivity, and substantial economic losses (12). IBR is a highly contagious viral disease caused by bovine herpesvirus-1 (BoHV-1) and causes respiratory and reproductive problems. Bovine brucellosis is a contagious bacterial disease mainly caused by *Brucella abortus* and occasionally by *Brucella melitensis* and *Brucella suis* and characterized by late-term abortion, loss of production, and zoonotic infections (13). In Ethiopia, IBR has progressively become widespread and a major challenge to the growing dairy industry. Previous studies reported IBR seroprevalence ranging from 25.6 to 79.1% in Ethiopia (3, 4, 12). Bovine brucellosis is a widespread and endemic animal disease in Ethiopia. Studies conducted on bovine brucellosis in the last two decades in Ethiopia indicated seroprevalence of the disease up to 22.5% at the animal level and up to 68.8% at the herd level (5, 8, 14).

In Ethiopia, dairy production has undergone extensive development and has become the source of food, income, and entrepreneurship contributing significantly to food security in the country. Herewith, in North Shewa, in the central highlands of Ethiopia, there is rapid growth and expansion of both smallholder and commercial dairy production. North Shewa, particularly Angolela Tera and Kimbibit districts, is well-known for producing highly demanded dairy products and is one of the major milkshed areas of the country. However, reproductive health problems are major constraints of smallholder and commercial dairy production in the country, which caused substantial economic losses due to reduced production, replacement herds, culling of potentially used cows, and infertility. In Ethiopia, a high prevalence of reproductive health problems including abortion, RFM, postpartum anoestrus (PPA), repeat breeding, and infectious diseases such as IBR and bovine brucellosis are widely occurring in commercial and smallholder dairy production (5, 10, 15–17). However, “limited information is available on the epidemiology of these reproductive health problems and their associated infectious diseases.” The identification of major reproductive disorders is of paramount importance for improving commercial and smallholder dairy production. Thus, this study was designed to identify the major reproductive health problems, associated infectious diseases, and risk factors in commercial and smallholder dairy herds in North Shewa, central highlands of Ethiopia.

## 2 Materials and methods

### 2.1 Description of the study areas

**Figure 1:**
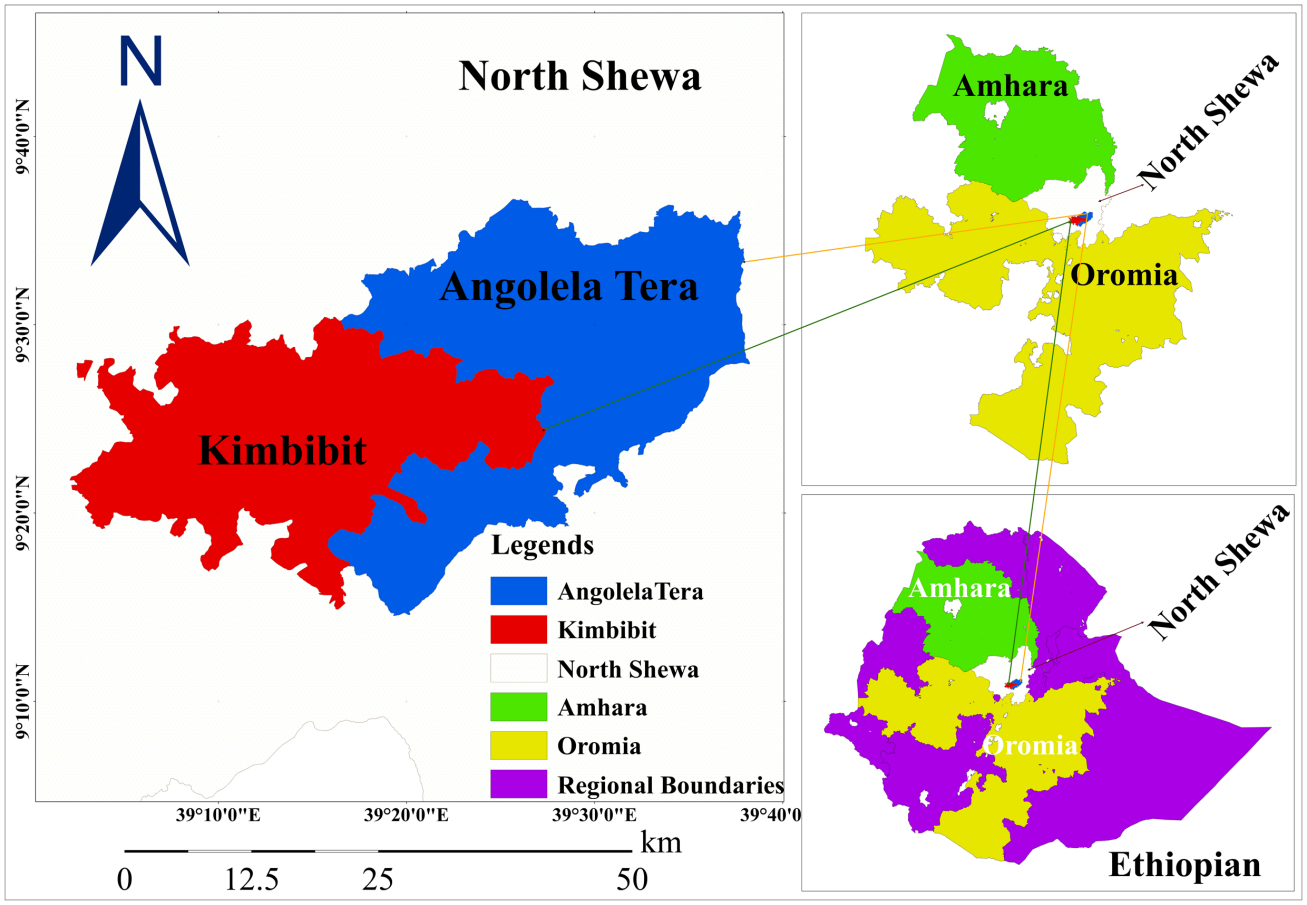
Map of the study areas in North Shewa zones of Amhara and Oromia regional states, central highlands of Ethiopia (Projected using ArcGIS software).

The study was conducted in selected districts of North Shewa in the central highlands of Ethiopia. This study primarily focused on the areas situated between the capital Addis Ababa and Debre Berhan metropolitan city (Figure 1). North Shewa represents one of the highest dairy-producing areas in Ethiopia. North Shewa is characterized by potential livestock production in the mixed crop-livestock production system with 1,704,407 heads of cattle, and dairy production is rapidly growing due to the climatic suitability and proximity to the country’s main cities (18). There is a wide expansion of commercial dairy industries, whereas smallholder dairy productions progressively increase with the introduction of improved and crossbred dairy cattle. The region is well known for producing and marketing highly demanded organic dairy products (5, 19).

Angolela Tera and Kimbibt districts were selected from the North Shewa zones of the Amhara and Oromia regional states, respectively. Angolela Tera district is geographically situated between 9°23′–9°60′N latitude and 39°26′–39°64′E longitude (Figure 1). The area was characterized by mean annual minimum and maximum temperatures of 6.7°C and 19.9°C, respectively. The annual rainfall in the district ranges from 800 to 1,500 mm. The district is majorly represented by highland and midland agro-ecologies with an altitude that ranges from 1,700 to 3,400 m above sea level (5). Kimbibit district is geographically extending between 9°12′–9°32′N latitude and 39°21′–39°33′E longitudes with an elevation range from 1,390 to 2,980 m above sea level (Figure 1). The climatic condition of the district is predominantly semi-arid climate. The mean annual rainfall is 913 mm, and the mean annual minimum and maximum temperature are 13°C and 19°C, respectively (20).

### 2.2 Study animals

The present study was conducted on dairy herds in commercial and smallholder dairy productions. Many small and large-scale dairy farmings in North Shewa, particularly in Angolela Tera and Kimbibit districts, supply milk and dairy products to Addis Ababa, Debre Berhan, and surrounding areas. The study dairy herds were composed of different cattle breeds, management systems, herd size, breeding methods, and herd replacement strategies. Indigenous cattle breeds and crosses of locals with Jersey and Holstein Friesians were the major cattle breeds in the study dairy herds. Dairy herd management includes intensive farming in commercial dairy farms, semi-intensive management systems in commercial and smallholder dairy herds, and extensive management systems in smallholder dairy production. The breeding strategies used in the dairy herds were categorized as natural (bull) mating, artificial insemination (AI), and dairy herds used both breeding methods. Herd replacement strategies were identified as dairy farms raising their replacement, purchasing replacement herds, and employing both strategies. Dairy herd size classifications were based on slight modifications of Alehegn et al. (21) into ≤5 cattle, 6–10 cattle, 11–20 cattle, and >20 cattle herd sizes (21).

### 2.3 Study design

A cross-sectional study was conducted on major reproductive health problems and associated infectious diseases from November 2022 to May 2023 in the selected districts of North Shewa. The study employed simultaneous research approaches based on laboratory investigation and field survey. Blood samples were collected to determine herd prevalence of infectious reproductive diseases. Retrospective data were collected to identify the major reproductive disorders that occurred in the last 2 years in both commercial and smallholder dairy herds. The potential risk factors include herd management systems, cattle breeds in dairy herds, herd replacement strategies, breeding methods, and herd sizes, which were collected during sample collection.

### 2.4 Sampling methodology and sample size determination

The study employed stratified random sampling to select dairy herds from commercial and smallholder dairy productions. The stratification was based on the farm types of the study dairy herds. Herewith, the study dairy herds were initially classified into commercial and smallholder dairy farms, and dairy herds were randomly selected from each farm type. The study districts were purposely selected as Angolela Tera and Kimbibit districts are the potential dairy-producing areas and are the major milkshed areas in the North Shewa zones of Amhara and Oromia regions, respectively (5). The sample size required in this study was determined based on Thrusfield (22) at a 95% level of confidence and 5% desired precision. The number of animals to be sampled was determined using a 50% expected prevalence (22). Accordingly, the minimum sample size required for the current study was 384 animals. The number of herds needed for this study was computed based on the 6.2% herd prevalence of bovine brucellosis reported on dairy farms in Debre Birhan (5). Therefore, the number of herds required for this study was 90 herds. However, this study included 142 dairy herds and 511 animals, and a proportional number of dairy herds were taken from each farm type in each district.

### 2.5 Blood sample collection

Blood samples were collected to determine herd prevalence of infectious reproductive diseases, particularly IBR and bovine brucellosis. Infectious bovine rhinotracheitis and bovine brucellosis are major diseases in both commercial and smallholder dairy farms in different parts of the country (4, 12). About 10 mL of blood sample was collected from the jugular vein using a plain vacutainer tube (BD Vacutainer®, UK). Fresh needles were used for each animal to eliminate the risk of cross-contamination. Each blood sample was labeled and kept at room temperature overnight in a slanting position to allow clotting and serum separation. The serum samples were gently decanted into 1.8 mL of cryovials. Finally, the samples were transported in the icebox to the laboratory and preserved at −20°C for serological investigation of IBR and brucellosis at the Animal Health Institute (AHI), Sebeta, Ethiopia.

### 2.6 Retrospective data collection

Retrospective data were collected from herd record books, dairy owners, and herdsmen to determine the major reproductive health problems and potential risk factors in commercial and smallholder dairy farms. Data were collected on dairy production systems and practices such as herd size, cattle breeds, management systems, breeding methods, herd replacement strategies, and sharing of bulls, and were considered as the risk factors of reproductive health problems. In this study, 2 years of retrospective data were collected on the occurrence of major reproductive health problems, including cases of abortion, stillbirth, retained fetal membranes, dystocia, cervical and vaginal prolapses, repeat breeding, anoestrus, and calf mortality in the dairy herds. Abortion was perceived in the case of the termination of pregnancy and expulsion of the fetus before reaching the stage of viability (23). Stillbirth was considered when the cow delivered a dead, full-term fetus (23). Retained fetal membranes have been identified as the failure to expel fetal membranes within 24 h after parturition (24). The difficulty during labor was recorded as a case of Dystocia (5). Cervical and vaginal prolapses were taken when the cervix and/or vagina protruded out of the vulvar lips (17). Repeat breeding was considered when the cow cycled normally and failed to conceive after at least two successive inseminations (25). The cow that failed to return to oestrus by 60 days postpartum has been identified as PPA (25). Neonatal calf mortality was perceived when calves were born alive and died within the first month of life (26).

### 2.7 Serological tests

The serological investigation of IBR was conducted using blocking IBR-gB ELISA, while the investigation of bovine brucellosis was performed using RBPT and CFT tests. A blocking enzymatic immunosorbent assay (B-ELISA) was conducted to detect antibodies against glycoprotein-B (gB) of *bovine alphaherpesvirus-1* (BoHV-1) using ELISA kits (HerdChek® IBR/BHV-1 gB-Ab test, IDEXX IBR gB-Ab Test, The Netherlands). The diagnostic sensitivity was determined to be 99%, whereas its specificity was 99.7% (3, 27). The serological analysis was performed according to the manufacturer’s instructions. A Bio-Tek microplate reader (model EL312 from Bio-Tek Instruments Inc., Winooski, VT, USA) was used to measure the optical density (OD) value at 450 nm. Following the manufacturer’s guidelines, a blocking percentage of <45 was interpreted as negative, while a blocking percentage >55 was considered positive. Intermediate values were suspected of BoHV-1, but this study was not reported as positive.

A Rose Bengal plate test (RBPT) with a diagnostic sensitivity of 92.9% and specificity of 77.6% was used to screen serum samples for bovine brucellosis. RBPT is a plate agglutination test with the antigen of *B. abortus* strain 99. The test was performed according to the World Organization for Animal Health (WOAH) protocols (28) and the manufacturer’s procedure. The results were recorded and interpreted as the absence of agglutination, barely visible agglutination, fine agglutination, and coarse agglutination. Serum samples indicating any visible agglutination were taken as positive. Samples positive on the RBPT test were subjected to a complement fixation test (CFT). CFT remains the most commonly used test for the serological confirmation of *Brucella* infection and is recommended by the World Organization for Animal Health (29). The sensitivity of CFT is 81% and is a specific test (95–100%). The positive reactions were indicated by the sedimentation of sheep red blood cells and the absence of hemolysis, and the result was read by recoding the minimum hemolytic dose.

### 2.8 Data management and statistical analysis

The data generated in this study were entered and managed in a Microsoft Excel spreadsheet. The many attribute data records include results of field and laboratory investigations such as the prevalence of major reproductive disorders, infectious diseases, and associated risk factors. The data were cleaned and coded for statistical analysis. The data were transferred to and analyzed using R-statistical software version 4.1.2. Descriptive statistics were used to summarize the data, and cross-tabulations and frequency tables were constructed. Univariable logistic regression statistical analysis was conducted to indicate the association of reproductive health problems with putative risk factors and infectious reproductive diseases in the dairy herd. Multicollinearity was checked for all predictor variables with the Variance Inflation Factor (VIF). Finally, multivariable logistic regression analyses were conducted to determine the adjusted effect of risk factors. Receiver operator curve (ROC) analysis was used to evaluate the goodness of fit of the final regression model. The variables with a *p*-value of less than 0.05 at a 95% CI were considered statistically significant factors of reproductive health problems.

## 3 Results

### 3.1 Seroprevalence of infectious reproductive diseases

In the present study, out of 142 dairy herds, 121 were found positive for IBR, with at least one positive animal in the herd. The overall herd prevalence of IBR was 85.21% (95% CI: 78.28–90.21) in commercial and smallholder dairy herds in North Shewa in the central highlands of Ethiopia. The current study revealed a 3.52% (95% CI: 1.46–8.26) herd prevalence of bovine brucellosis in dairy animals in the study areas. IBR and bovine brucellosis were identified in both commercial and smallholder dairy herds. However, the seroprevalence results revealed that IBR is a significantly more prevalent infectious reproductive disease compared to bovine brucellosis (*p* = 0.000) in the study areas (Table 1).

**Table 1:** Seroprevalence of infectious reproductive diseases in commercial and smallholder dairy herds in the study areas.

Infectious diseases	No. of herds tested	Positive herds (%)	95% CI	*p*-value
IBR	142	121 (85.21)	78.28–90.21	0.000
Bovine brucellosis	142	5 (3.52)	1.46–8.26	

CI, Confidence interval.

### 3.2 Major reproductive health problems in dairy herds

**Figure 2:**
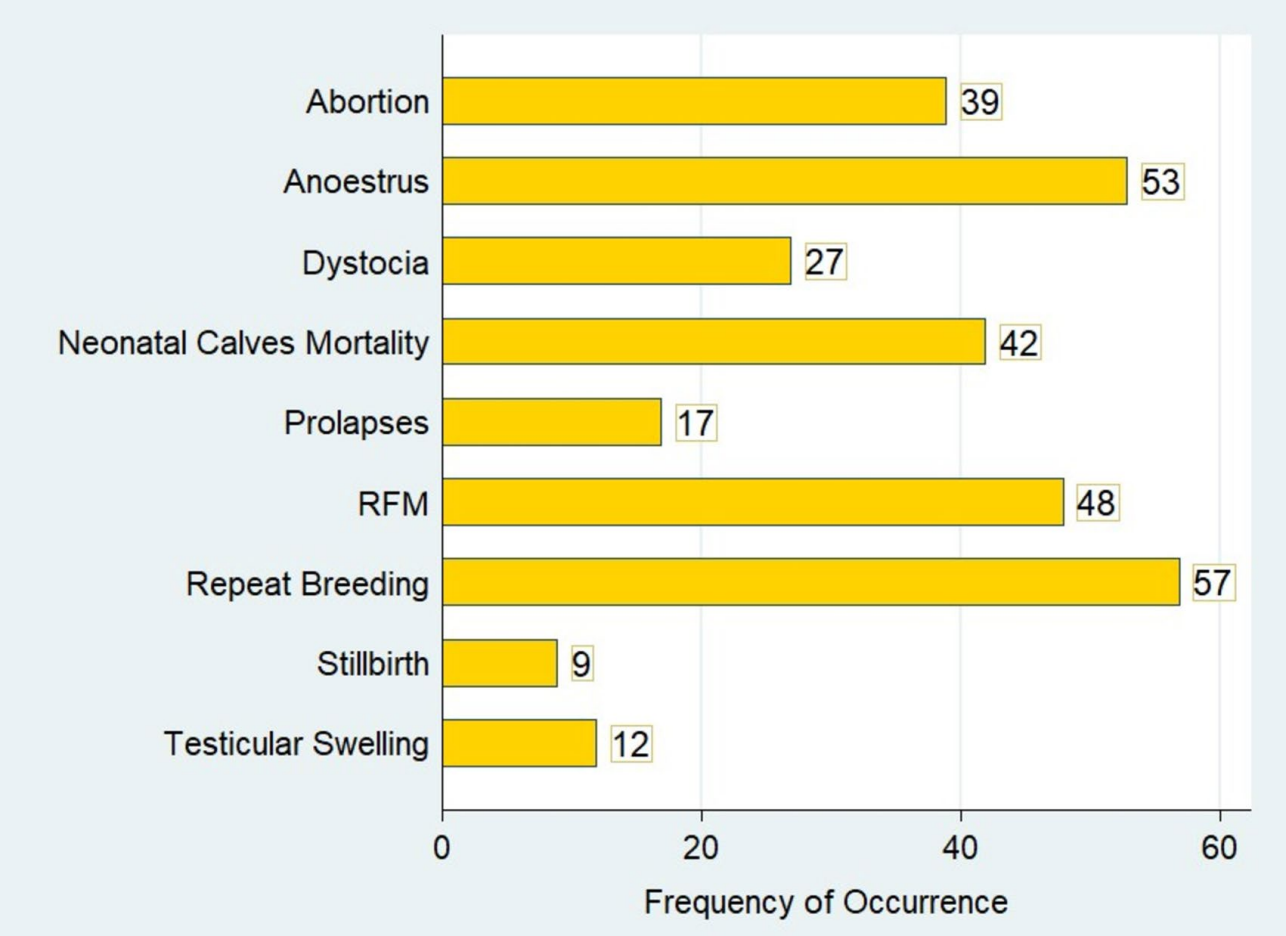
Graphic illustration of the major reproductive health problems in dairy herds in the study areas.

This study identified higher reproductive health problems in dairy herds in the central highlands of Ethiopia. Out of 142 commercial and smallholder dairy herds, abortion was observed in 39 (27.46%; 95% CI: 20.68–35.48) dairy herds (Figure 2). The prevalence of retained placenta was 33.80% (95% CI: 26.42–42.06), whereas the prevalence of dystocia in the herds was 19.01% (95% CI: 13.32–26.41). The herd prevalence of cervical and vaginal prolapse was 12% (95% CI: 7.53–18.51) in the dairy herds. In the present study, stillbirth was the least prevalent reproductive problem and was identified in only 9 (6.34%; 95% CI: 3.30–11.82) dairy herds in the North Shewa (Table 2).

**Table 2:** The prevalence of major reproductive health problems in commercial and smallholder dairy herds.

Reproductive health problems	Total no. of herds	Positive herds (%)	95% CI
Abortion	142	39 (27.46)	20.68–35.48
RFM	142	48 (33.80)	26.42–42.06
Dystocia	142	27 (19.01)	13.32–26.41
Stillbirth	142	9 (6.34)	3.30–11.82
Cervical and vaginal prolapses	142	17 (11.97)	7.53–18.51
Calf mortality	142	42 (29.58)	22.58–37.69
Anoestrus	142	53 (37.32)	29.68–45.66
Repeat breeding	142	57 (40.14)	32.32–48.50
Testicular swelling	142	12 (8.45)	4.83–14.38

CI, Confidence interval.

Repeat breeding and anoestrus were the most prevalent reproductive health problems in both commercial and smallholder dairy herds in North Shewa. Repeat breeder cows were identified in 57 of 142 dairy herds (Figure 2). In this study, repeat breeding was the most prevalent reproductive problem, with 40.14% (95% CI: 32.32–48.50) herd prevalence in the dairy herds. Anoestrus was determined in 37.32% (95% CI: 29.68–45.66) of dairy herds and found to be the second most prevalent reproductive health problem. Neonatal calf mortality was found to be one of the most important reproductive health problems, with a prevalence of 29.58% (95% CI: 22.58–37.69) in commercial and smallholder dairy herds (Table 2).

### 3.3 Prevalence of abortion and associated risk factors

The present study revealed the occurrence of abortion was significantly associated with infectious reproductive diseases. The occurrence of abortion was 8 (OR = 8.34; 95% CI: 1.91–76.96; *p* = 0.006) times higher in dairy herds with IBR prevalence. Accordingly, the prevalence of brucellosis increased the risk of abortion by 21 (95% CI: 1.80–421.84; *p* = 0.037) times in the herd. The multivariable logistic regression analysis indicated that as the herd size increased, there was a higher probability of the occurrence of abortion in the herd. Herds with >20 herd size were 25.47 (95% CI: 1.49–436.50; *p* = 0.026) times more observed with abortion, whereas herds with 11–20 herd size were 3.23 (95% CI: 1.67–15.48; *p* = 0.043) times at a higher risk of abortion as compared to herds with ≤5 herd size (Table 3).

**Table 3:** Univariable and multivariable logistic regression analysis on the prevalence of abortion and associated risk factors in dairy herds.

Predictive variables	No. of herds	Herds with abortion (%)	Crude OR (95% CI)	*p*-value	Adjusted OR (95% CI)	*p*-value
IBR						
Negative	21	1 (4.76)	1		1	
Positive	121	38 (31.40)	9.16 (1.19–70.75)	0.034	8.34 (1.91–76.96)	0.006
Brucellosis						
Negative	137	34 (24.82)	1		1	
Positive	5	5 (100)	33.00 (1.78–612.25)	0.019	21.07 (1.80–421.84)	0.037
Cattle breed						
Local	36	7 (19.44)			1	
Cross	33	9 (27.27)	1.55 (0.50–4.79)	0.443	0.80 (0.17–3.76)	0.774
Both	73	23 (31.51)	1.91 (0.73–4.99)	0.189	1.60 (0.52–4.89)	0.408
Herd size						
≤5	27	4 (14.81)	1		1	
6–10	73	14 (19.18)	1.36 (0.41–4.58)	0.615	1.21 (0.31–4.79)	0.782
11–20	33	13 (39.39)	3.74 (1.05–13.32)	0.042	3.23 (1.67–15.48)	0.043
>20	9	8 (88.89)	46 (4.46–474.83)	0.001	25.47 (1.49–436.50)	0.026
Production system						
Extensive	122	30 (24.59)	1		1	
Semi-intensive	12	6 (50.00)	3.07 (0.92–10.23)	0.068	0.93 (0.10–9.10)	0.954
Intensive	8	3 (37.50)	1.84 (0.41–8.16)	0.422	0.17 (0.01–3.29)	0.240
Replacement strategy						
Own	27	10 (37.04)			1	
Purchased	6	5 (83.33)	8.5 (0.87–83.49)	0.066	4.50 (0.07–273.47)	0.473
Both	109	24 (22.02)	0.48 (0.19–1.18)	0.111	0.37 (0.12–1.09)	0.071
Breeding method						
AI	24	2 (8.33)	1		1	
Bull	91	28 (30.77)	4.89 (1.08–22.23)	0.040	4.58 (1.71–29.69)	0.011
Both	27	9 (33.33)	5.5 (1.05–28.75)	0.043	5.28 (1.85–32.75)	0.047
Sharing of bulls						
Absent	62	11 (17.74)	1		1	
Present	80	28 (35.00)	2.50 (1.12–5.54)	0.025	1.98 (0.30–3.21)	0.077

OR, Odds ratio; 1, Reference category; CI, Confidence interval.

In this study, abortion was found to be significantly associated with breeding methods used in the herd. The prevalence of abortion was 5 (95% CI: 1.71–29.69; *p* = 0.011) times higher in dairy herds using natural or bull mating systems and 5.28 (95% CI: 1.85–32.75; *p* = 0.047) times higher in herds employing both natural mating and AI systems than in herds using only AI systems. The presence of group mating and sharing of bulls in the herds was found to be a significant factor in the prevalence of abortion on the univariable logistic regression model (OR = 2.50; 95% CI: 1.12–5.54; *p* = 0.025). Herd-level factors, including cattle breeds, production systems, and replacement strategies, were less important factors for abortion (Table 3).

### 3.4 Retained fetal membrane (RFM) and associated risk factors

The multivariable logistic regression model indicated that the prevalence of infectious reproductive diseases, production systems, herd replacement strategies, and the presence sharing of bulls between herds were significantly associated with RFM in the herd. Herds with a prevalence of IBR were 9 (95% CI: 1.04–70.89; *p* = 0.045) times more exposed to RFM, whereas herds with brucellosis were 19 (95% CI: 1.67–256.77; *p* = 0.003) times more at risk of RFM. The prevalence of retained placenta was 4 (95%CI: 1.56–28.67; *p* = 0.016) times higher in herds kept under intensive management systems than in herds kept under extensive production systems. Herds that used purchased replacements were more exposed to RFM (OR = 16.21; 95% CI: 2.42–324.88, *p* = 0.006) as compared to herds that raised their replacements. The present study revealed that the presence of bulls sharing among dairy herds aggravates the risk of RFM. Herds that shared bulls for mating were 4 (95% CI: 1.12–13.74; *p* = 0.033) times more at risk of retained placenta than herds that used their bulls (Table 4).

**Table 4:** Univariable and multivariable logistic regression analysis of RFM prevalence and associated risk factors in dairy herds.

Predictive variables	No. of herds	Herds with RFM (%)	Crude OR (95% CI)	*p*-value	Adjusted OR (95% CI)	*p*-value
IBR						
Negative	21	2 (9.52)	1		1	
Positive	121	46 (38.02)	5.83 (1.30–26.18)	0.022	8.61 (1.04–70.89)	0.045
Brucellosis						
Negative	137	43 (31.39)	1		1	
Positive	5	5 (100)	23.90 (1.29–441.88)	0.033	19.07 (1.67–256.77)	0.003
Cattle breed						
Local	36	10 (27.78)	1		1	
Cross	33	13 (39.39)	1.69 (0.62–4.64)	0.308	1.11 (0.29–4.25)	0.876
Both	73	25 (34.25)	1.35 (0.56–3.25)	0.497	1.30 (0.48–3.56)	0.607
Herd size						
≤5	27	6 (22.22)	1		1	
6–10	73	19 (26.03)	1.23 (0.43–3.51)	0.697	0.88 (0.26–2.98)	0.841
11–20	33	16 (48.48)	3.29 (1.06–10.25)	0.040	1.29 (0.31–5.38)	0.731
>20	9	7 (77.78)	12.25 (1.20–75.20)	0.007	1.80 (0.18–18.49)	0.621
Production system						
Extensive	122	35 (28.69)	1		1	
Semi-intensive	12	7 (58.33)	3.48 (1.03–11.70)	0.044	2.25 (0.42–12.03)	0.344
Intensive	8	6 (75.00)	7.45 (1.44–38.74)	0.017	4.01 (1.56–28.67)	0.016
Replacement strategy						
Own	27	8 (29.63)	1		1	
Purchased	6	6 (100)	29.82 (1.50–591.38)	0.026	16.21 (2.42–324.88)	0.006
Both	109	34 (31.19)	1.08 (0.43–2.70)	0.875	1.05 (0.38–2.94)	0.914
Breeding method						
AI	24	7 (29.17)	1		1	
Bull	91	30 (32.97)	1.19 (0.45–3.19)	0.723	0.34 (0.08–1.50)	0.155
Both	27	11 (40.74)	1.70 (0.52–5.37)	0.390	0.91 (0.24–3.50)	0.896
Sharing of bulls						
Absent	62	14 (22.58)	1		1	
Present	80	34 (42.50)	2.53 (1.21–5.32)	0.014	3.92 (1.12–13.74)	0.033

OR, Odds ratio; 1, Reference category; CI, Confidence interval.

### 3.5 Repeat breeding and associated risk factors in dairy herds

In this study, the prevalence of repeat breeders in the herds was significantly associated with cattle breeds in the herd, herd size, breeding methods, the presence of bull sharing among herds, and the prevalence of IBR. The risk of repeat breeding was increased by 3.16 (95% CI: 1.82–12.23; *p* = 0.009) times in herds exposed to IBR infection. This study indicated that bovine brucellosis did not significantly correlate with repeat breeding in dairy herds (*p* > 0.05). The prevalence of repeat breeders was 3.62 (95% CI: 1.91–14.37; *p* = 0.006) times higher in herds constituting only crossbred cattle than in herds with only local cattle breeds. In the univariable model, there were significant differences in the prevalence of repeat breeders among herd management systems (Table 5).

**Table 5:** Univariable and multivariable logistic regression model on the prevalence of repeat breeding and associated risk factors.

Predictive variables	No. of herds	Herds with repeat breeder (%)	Crude OR (95% CI)	*p*-value	Adjusted OR (95% CI)	*p*-value
IBR						
Negative	21	4 (19.05)	1		1	
Positive	121	53 (43.80)	3.31 (1.05–10.43)	0.041	3.16 (1.82–12.23)	0.009
Brucellosis						
Negative	137	52 (37.96)	1		1	
Positive	5	5 (100)	17.91 (0.97–330.64)	0.052	12.26 (0.86–242.36)	0.058
Cattle breed						
Local	36	8 (22.22)	1		1	
Cross	33	18 (54.55)	4.2 (1.48–11.91)	0.007	3.62 (1.91–14.37)	0.006
Both	73	31 (42.47)	2.58 (1.04–6.43)	0.042	2.77 (0.911–8.41)	0.073
Herd size						
≤5	27	8 (29.63)	1		1	
6–10	73	21 (28.77)	0.95 (0.36–2.53)	0.933	0.52 (0.17–1.64)	0.267
11–20	33	19 (57.58)	3.22 (1.10–9.46)	0.033	1.87 (0.23–3.29)	0.835
>20	9	9 (100)	43.59 (2.27–837.61)	0.012	18.66 (2.66–425.28)	0.032
Production system						
Extensive	122	42 (34.43)	1		1	
Semi-intensive	12	9 (75.00)	5.71 (1.47–22.24)	0.012	1.52 (0.25–9.28)	0.648
Intensive	8	6 (75.00)	5.71 (1.10–29.56)	0.038	3.12 (0.34–28.68)	0.314
Replacement strategy						
Own	27	13 (48.15)	1		1	
Purchased	6	5 (83.33)	5.38 (0.55–52.43)	0.147	1.15 (0.05–28.82)	0.930
Both	109	39 (35.78)	0.6 (0.26–1.40)	0.239	0.52 (0.19–1.44)	0.207
Breeding method						
AI	24	13 (54.17)	1		1	
Bull	91	28 (30.77)	0.37 (0.15–0.94)	0.037	0.11 (0.02–0.47)	0.003
Both	27	16 (59.26)	1.23 (0.40–3.74)	0.714	0.92 (0.26–3.27)	0.895
Sharing of bulls						
Absent	62	24 (38.71)	1		1	
Present	80	33 (41.25)	1.11 (0.56–2.19)	0.759	3.84 (1.05–14.12)	0.043

OR, Odds ratio; 1, Reference category; CI, Confidence interval.

The current study dictated that the prevalence of repeat breeding in the herd was 19 (OR = 18.66; 95% CI: 2.66–425.28; *p* = 0.032) times higher in herds with greater than 20 herd sizes as compared to herds ≤5 herd sizes. There was a significant association between breeding methods and repeat breeding in the herd. The prevalence of repeat breeders was significantly lower (OR = 0.11; 95% CI: 0.02–0.47; *p* = 0.003) in herds using the natural mating system than in herds using the AI system. However, the risk of repeat breeding was increased by 3.84 (95% CI: 1.05–14.12; *p* = 0.043) times in herds that shared bulls for mating as compared to herds using their breeding bulls (Table 5).

### 3.6 Prevalence of anoestrus and associated risk factors

The present study revealed the significant association of anoestrus with IBR, brucellosis, cattle breeds, herd size, herd replacement strategies, and the presence of bull sharing for mating. The prevalence of anoestrus was 7 (95% CI: 1.28–34.38; *p* = 0.024) times higher in herds with IBR prevalence, whereas herds with brucellosis infection were 12.48 (95% CI: 1.63–182.69; *p* = 0.002) times more at risk of anoestrus in dairy herds. In this study, the prevalence of anoestrus was lower in crossbred cattle (OR = 0.06; 95% CI: 0.01–0.82; *p* = 0.009) as compared to local cattle breeds. Herds with 6–10 cattle sizes were less exposed to anoestrus (OR = 0.11; 95% CI: 0.02–0.46; *p* = 0.003) than herds with ≤5 herd sizes. There was a significant difference in the prevalence of anoestrus among herd replacement strategies. Anoestrus was 17 (95% CI: 2.31–124.66; *p* = 0.009) and 6 (95% CI: 1.62–20.89; *p* = 0.007) times higher in herds that used purchased replacements and in herds that employed both replacement strategies, respectively, compared to herds that raised their replacements. The presence of group mating or sharing of bulls between herds increased the risk of anoestrus by 7.24 (95% CI: 1.38–38.01; *p* = 0.019) times in dairy herds (Table 6).

**Table 6:** Univariable and multivariable logistic regression analysis of anoestrus prevalence and associated risk factors.

Predictive variables	No. of herds	Herds with anoestrus (%)	Crude OR (95% CI)	*p*-value	Adjusted OR (95% CI)	*p*-value
IBR						
Negative	21	3 (14.29)	1		1	
Positive	121	50 (41.32)	4.23 (1.18–15.12)	0.027	6.63 (1.28–34.38)	0.024
Brucellosis						
Negative	137	48 (35.04)	1		1	
Positive	5	5 (100)	20.30 (1.10–374.91)	0.043	12.48 (1.63–182.69)	0.002
Cattle breed						
Local	36	13 (36.11)	1		1	
Cross	33	4 (12.12)	0.24 (0.07–0.85)	0.027	0.06 (0.01–0.82)	0.009
Both	73	36 (49.32)	1.72 (0.76–3.91)	0.194	1.77 (0.60–5.18)	0.301
Herd size						
≤5	27	11 (40.74)	1		1	
6–10	73	20 (27.40)	0.55 (0.22–1.38)	0.203	0.11 (0.02–0.46)	0.003
11–20	33	15 (45.45)	1.21 (0.43–3.39)	0.714	0.50 (0.09–2.88)	0.437
>20	9	7 (77.78)	5.09 (0.89–29.27)	0.068	2.81 (0.92–12.56)	0.991
Production system						
Extensive	122	43 (35.25)	1		1	
Semi-intensive	12	5 (41.67)	1.31 (0.39–4.38)	0.659	0.28 (0.02–4.20)	0.357
Intensive	8	5 (62.50)	3.06 (0.70–13.43)	0.138	3.53 (0.18–68.47)	0.405
Replacement strategy						
Own	27	5 (18.52)	1		1	
Purchased	6	5 (83.33)	21.99 (2.08–232.16)	0.010	16.91 (2.31–124.66)	0.009
Both	109	43 (39.45)	2.87 (1.01–8.14)	0.048	5.82 (1.62–20.89)	0.007
Breeding method						
AI	24	8 (33.33)	1		1	
Bull	91	32 (35.16)	1.08 (0.42–2.81)	0.867	1.10 (0.01–1.69)	0.230
Both	27	13 (48.15)	1.86 (0.60–5.78)	0.285	1.35 (0.25–7.37)	0.733
Sharing of bulls						
Absent	62	16 (25.81)	1		1	
Present	80	37 (46.25)	2.47 (1.21–5.08)	0.014	7.24 (1.38–38.01)	0.019

OR, Odds ratio; 1, Reference category; CI, Confidence interval.

### 3.7 Neonatal calf mortality and associated risk factors in dairy herds

In the present study, neonatal calf mortality was significantly influenced by infectious diseases such as IBR, herd size, breeding methods, and sharing of bulls among herds. The risk of calf mortality was 4 (95% CI: 1.81–20.32; *p* = 0.008) times higher in herds infected with IBR than in herds free from IBR. Bovine brucellosis infection in herds showed a significant effect on calf mortality in the univariable logistic regression model. The present study indicated a higher prevalence of calf mortality in herds as the size of the herd increased. Herds with 11–20 herd sizes were 6.39 (95% CI: 1.42–28.74; *p* = 0.016) times more exposed to calf mortality, while herds with >20 herd sizes were 32.84 (95% CI: 1.96–551.36; *p* = 0.015) times at higher risk of neonatal calf mortality than herds with ≤5 herd sizes (Table 7).

**Table 7:** Univariable and multivariable logistic regression analysis of neonatal calf mortality and associated risk factors in dairy herds.

Predictive variables	No. of herds	Herds with calf mortality (%)	Crude OR (95% CI)	*p*-value	Adjusted OR (95% CI)	*p*-value
IBR						
Negative	21	2 (9.52)	1		1	
Positive	121	40 (33.06)	4.69 (1.04–21.14)	0.044	4.05 (1.81–20.32)	0.008
Brucellosis						
Negative	137	38 (27.74)	1		1	
Positive	5	4 (80.00)	10.42 (1.13–96.24)	0.039	0.97 (0.03–26.98)	0.984
Cattle breed						
Local	36	9 (25.00)	1		1	
Cross	33	12 (36.36)	1.71 (0.61–4.83)	0.308	0.76 (0.19–3.14)	0.708
Both	73	21 (28.77)	1.21 (0.49–3.01)	0.679	0.93 (0.32–2.76)	0.899
Herd size						
≤5	27	5 (18.52)	1		1	
6–10	73	14 (19.18)	1.04 (0.34–3.24)	0.941	1.47 (0.40–5.39)	0.558
11–20	33	15 (45.45)	3.67 (1.12–12.03)	0.032	6.39 (1.42–28.74)	0.016
>20	9	8 (88.89)	35.20 (3.55–349.15)	0.002	32.84 (1.96–551.36)	0.015
Production system						
Extensive	122	30 (24.59)	1		1	
Semi-intensive	12	7 (58.33)	4.29 (1.27–14.53)	0.019	2.45 (0.35–17.08)	0.366
Intensive	8	5 (62.50)	5.11 (1.15–22.67)	0.032	1.72 (0.23–12.87)	0.595
Replacement strategy						
Own	27	8 (29.63)	1		1	
Purchased	6	4 (66.67)	4.75 (0.72–31.37)	0.106	1.58 (0.06–43.89)	0.789
Both	109	30 (27.52)	0.90 (0.36–2.28)	0.827	0.82 (0.25–2.64)	0.738
Breeding method						
AI	24	2 (8.33)	1		1	
Bull	91	31 (34.07)	5.68 (1.25–25.75)	0.024	17.01 (2.56–113.09)	0.003
Both	27	9 (33.33)	5.50 (1.05–28.75)	0.043	5.52 (0.85–35.73)	0.073
Sharing of bulls						
Absent	62	17 (27.42)	1		1	
Present	80	25 (31.25)	1.20 (0.58–2.50)	0.620	0.24 (0.08–0.77)	0.016

OR, Odds ratio; 1, Reference category; CI, Confidence interval.

The multivariable logistic regression model stated that herds that used the bull mating system were 17 (95% CI: 2.56–113.09; *p* = 0.003) times more exposed to neonatal calf mortality than herds that used AI systems. Accordingly, the univariable logistic regression analysis indicated a significant difference in the prevalence of calf mortality among herd management systems. The present study revealed a significant association between calf mortality and the presence of group mating or the sharing of bulls for mating. The prevalence of neonatal calf mortality was significantly lower (OR = 0.24; 95% CI: 0.08–0.77; *p* = 0.016) in herds that shared bulls for mating than in herds that used their breeding bulls (Table 7).

## 4 Discussion

The present study revealed the occurrence of various reproductive health problems and the widespread distribution of infectious reproductive diseases in commercial and smallholder dairy herds in North Shewa in the central highlands of Ethiopia. In the current study areas, IBR was prevalent in 85% (95% CI: 78.28–90.21) of dairy herds. Similarly, studies conducted in Ethiopia and other parts of the world reported similar findings. Studies reported IBR herd prevalence of 81.8% in central, southern, and southwestern milkshed areas of Ethiopia (16), 82.1% in non-vaccinated dairy and dual-purpose cattle herds in Ecuador (30), and 94% in dairy herds in central Costa Rica (6). The higher prevalence of IBR in this study could be due to the lack of integrated IBR control measures, such as vaccination, herd screening and culling, and biosecurity measures in Ethiopia (3, 31).

The herd prevalence of bovine brucellosis (3.52%; 95% CI: 1.46–8.26) in the current study was in close agreement with studies that reported brucellosis prevalences of 4.76% in the Jimma zone (32), 5.8% in the Wolaita zone (33), and 4.8% in southern Oman (34). However, several studies reported a higher herd prevalence of bovine brucellosis in Ethiopia and across the world, including 12.3% in western Ethiopia (14), 33.33% in South Africa (35), and 21.14% in Zambia (36). The relatively lower prevalence of brucellosis in dairy herds in this study is supposed to be due to the holding of small herd sizes in mixed crop-livestock production, lesser communal grazing and herd mixing, and restricted animal movements in most central highlands of Ethiopia (37).

The current study indicated the prevalence of abortion in 27.46% (95% CI: 20.68–35.48) of dairy herds. The finding in this study was in agreement with herd abortion of 27% in Debre Berhan (5) and 28.30% in the central Tigray region (17). In contrast, some scholars reported a lower herd prevalence of abortion (10, 38, 39). The widespread distribution of infectious diseases, including IBR and brucellosis, higher dairy production, and management practices might cause the higher prevalence of abortion in this study. The present study revealed a significant association between the prevalence of abortion and infectious reproductive diseases. Dairy herds exposed to IBR were 8 (95% CI: 1.91–76.96; *p* = 0.006) times more at risk of abortion. Similarly, IBR was reported as a significant cause of abortion in dairy herds in different countries (12, 40, 41). Accordingly, herds infected with brucellosis were 21 (95% CI: 1.80–421.84; *p* = 0.037) times at higher risk of abortion. In agreement, a significant association between abortion and brucellosis was reported in dairy cows in Holeta in Ethiopia (42) and in dairy cattle in Indonesia (43).

The multivariable logistic regression analysis revealed a significant association of abortion with herd sizes. Accordingly, the occurrence of abortion was increased in larger dairy herd sizes in Chilean dairy herds (44) and in cattle herds in Ireland (45). However, some findings (39, 46) reported insignificant associations of herd sizes with abortion. The variations in the findings might arise from differences in herd classification and herd management practices. The significant effect of herd sizes on abortion in this study suggested higher herd-level abortion in larger herd sizes and abortifacient diseases rather than biological associations. Abortion was significantly influenced by breeding methods used in the herd, where herds using natural mating systems had higher exposure to abortion than herds using AI systems. In line with this, Tolosa et al. (10) reported a significant association of abortion with mating systems in dairy herds. This might infer the potential transmission of infectious diseases like brucellosis and IBR via bulls. In contrast, Tulu and Negera (39) indicated insignificant associations of abortion with breeding methods.

This study revealed a 33.80% (95% CI: 26.42–42.06) prevalence of retained placental membrane in dairy herds. In accordance, Weldegebriall (17) reported a 35.8% RFM in dairy cattle in the central zone of the Tigray region. However, a study conducted on dairy cattle in Hawassa town reported 13.2% of RFM (38). The differences in herd management practices, reproductive diseases, or retrospective data collection are supposed to be the underlying reasons for the variations in the findings. The present study indicated significant associations of RFM with IBR, brucellosis, production systems, herd replacement strategies, and sharing of bulls for mating. Similarly, several scholars (12, 16, 42, 47) indicated the significant association of RFM with infectious reproductive diseases. Accordingly, studies conducted in Bale Robe (10), Jimma Zone (48), and northwest Ethiopia (49) revealed significant differences in the prevalence of RFM with production systems, breeding methods, and herd management practices. The present finding indicated the inevitable role of infectious diseases and management practices as causes of RFM in dairy cattle herds.

Repeat breeding was the most prevalent reproductive health problem in dairy herds in the current study areas. Repeat breeders were found in 40.14% (95% CI: 32.32–48.50) of dairy herds. Similarly, findings reported repeat breeding in 38.4% of dairy herds in the northern highlands of Ethiopia (15) and 26.8% of animals in Assella town (50). The present study revealed a significant association between repeat breeding and IBR and that herds infected with IBR were more than 3 times more exposed to repeat breeding. In line with this study, several findings (12, 31, 51) indicated a significant effect of IBR on repeat breeding. In this study, brucellosis was found to be a less important factor in repeat breeding. In contrast, brucellosis was indicated as a significant factor of repeat breeding in dairy cattle in Bangladesh (52). Herds that shared bulls for mating were four times at a higher risk of repeat breeding. The findings signify sharing of bulls and group mating increases the transmission of reproductive diseases that cause several reproductive problems, such as repeat breeding in dairy herds (39).

The current study demonstrated the significant associations of repeat breeding with cattle breeds, herd sizes, and breeding methods. The prevalence of repeat breeders was higher in herds with crossbred cattle, larger herd sizes, and artificial insemination systems. Herds composed of crossbred cattle were 4 times more exposed to repeat breeders than herds with local cattle breeds. Similarly, Abaya et al. (48) showed crossbred cattle were more at risk of repeat breeding than local cattle breeds. Higher exposure of herds composed of crossbred cattle to repeat breeding could be attributed to infectious diseases, breeding methods, and management practices. Accordingly, Eshete et al. (15) indicated a higher repeat breeding in herds using AI systems and herds with large herd sizes. The higher rates of repeat breeding in herds that used AI systems suggested the lower efficiency of AI, insemination timing, and technical limitations (25).

In the present study, anoestrus was prevalent in 37.32% (95% CI: 29.68–45.66) of dairy herds. In line with this finding, the prevalence of anoestrus was 38.6% in the central highlands of Ethiopia (23) and 20% in central Tigray (17). The significant associations between anoestrus and infectious diseases were in line with several findings (12, 52). Herds infected with IBR and bovine brucellosis were exposed to anoestrus 7 and 12 times more, respectively. Infection of reproductive tracts with infectious diseases leads to postpartum anoestrus and infertility problems. Furthermore, anoestrus was significantly associated with cattle breeds, herd replacement strategies, and sharing of bulls for mating. Similarly, several findings (53–55) indicated a significant effect of cattle breeds, replacement strategies, and herd management practices on the occurrence of anoestrus, in particular PPA. The findings emphasized the inevitable impact of diseases and mismanagement practices on delaying and impairing the normal estrus cycle in dairy herds.

Neonatal calf mortality was among the most important reproductive problems and constraints of dairy production in the current study areas. The present study revealed the prevalence of calf mortality in 29.58% (95% CI: 22.58–37.69) of dairy herds. Similarly, 29.1–39.8% of calf mortality was reported in cattle and buffalo in India (26). However, Mee (56) reported 8% calf mortality at the farm level in Ireland. This study indicated a significant association of neonatal calf mortality with IBR. Accordingly, infectious diseases were indicated as the major cause of calf mortality (56, 57). The prevalence of neonatal calf mortality was significantly associated with herd size and breeding methods. In line with this finding, several studies (26, 58, 59) showed significant impacts of herd management practices on calf mortality. The higher neonatal calf mortality in the current study areas might be seen due to the higher prevalence of infectious diseases such as IBR, weather conditions, calving seasons, and calf management practices (26).

## 5 Conclusion

The present study identified the widespread distribution of reproductive health problems and associated infectious diseases in commercial and smallholder dairy herds. Infectious bovine rhinotracheitis was detected in 85% of dairy herds, whereas bovine brucellosis was prevalent in 4% of dairy herds in North Shewa in the central highlands of Ethiopia. The most prevalent reproductive health problems were abortion, retained placenta, repeat breeding, anoestrus, and neonatal calf mortality. Stillbirth was identified as the least important reproductive health problem in the current study areas. The present study revealed that most reproductive health problems were significantly associated with infectious diseases. IBR was significantly associated with abortion, RFM, repeat breeding, anoestrus, and neonatal calf mortality, while brucellosis was associated with abortion, RFM, and anoestrus. The study identified cattle breed, herd size, production systems, breeding methods, sharing of bulls for mating, and herd replacement strategies as significant risk factors for reproductive health problems. Thus, this study suggests the implementation of integrated disease control measures such as vaccination against BoHV-1, strict biosecurity measures, herd screening and culling, and sound herd management practices. Further studies are required to determine the prevalence of additional reproductive diseases and synergistic co-infections and their impacts on the reproductive health of dairy cattle.

## Data Availability

The raw data supporting the conclusions of this article will be made available by the authors without undue reservation.
